# Supplementary material for: Accurate Sequence-Dependent Coarse-Grained Model for Conformational and Elastic Properties of Double-Stranded DNA
Source: J Chem Theory Comput. 2022 Apr 8;18(5):3239–56. doi: 10.1021/acs.jctc.2c00138 (PMC9097290; doi:10.1021/acs.jctc.2c00138)
Supplement: Supplementary file 1 — ct2c00138_si_001.pdf [file ct2c00138_si_001.pdf]

Accurate sequence-dependent coarse-grained model for conformational and elastic properties of double-stranded DNA

Salvatore Assenza [1,2] and Rubén Pérez[1,2]

[1]Departamento de Física Teórica de la Materia Condensada, Universidad Autónoma de Madrid, E-28049 Madrid, Spain and [2]Condensed Matter Physics Center (IFIMAC), Universidad Autónoma de Madrid, E-28049 Madrid, Spain

Supplementary Information

S1 Coarse-graining procedure

In MADna, six different moieties are considered as effective particles, corresponding to the sugar, the phosphate group and the four bases. The effective particles are located in the geometrical centers of the heavy atoms belonging to each moiety. Specifically, for a sugar particle the center is computed considering the atoms C1', C2', C3', C4', C5' and O4', while for the phosphate group the atoms are O3', P, OP1, OP2 and O5. The rest of heavy atoms within the nucleotide are assigned to the base. The size σ of each bead was fixed following Ref.[1]. The values are reported in Table S1.

Name	Abbreviation	σ (Å)	mass (g/mol)
Sugar	S	6.4	76.05
Phosphate group	P	4.5	94.97
Adenine	A	5.4	130.09
Cytosine	C	6.4	106.06
Guanine	G	4.9	146.09
Thymine	T	7.1	120.07

Table S1: Excluded volume size (σ) and mass of the various beads.

S2 Determination of the force-field parameters

S2.1 Input atomistic simulations

The force-field parameters were fixed by matching coarse-grained predictions to the atomistic results obtained for a set of sequences encompassing all the possible step combinations studied in Ref. [2] (Training Sequences). For benchmarking, we further considered an independent set of atomistic simulations, reported in Refs. [2, 3] (Testing Sequences). The corresponding sequences are listed in Section S3.1. For clarity, we report here the main details of the atomistic simulations. For further information, we refer the reader to the original publications [2, 3]. Each sequence was simulated under the action of an external pulling force. Five values were considered (1,5,10,15,20 pN) in independent microsecond-long simulations with explicit solvent, with a cubic simulation box of size ∼85 Å. The initial structures were obtained by using the software NAB[4], and the overall charge of the system was kept neutral by adding a suitable number of sodium counterions, without additional salt. A standard preparation protocol was implemented, where the system was sequentially subjected to an energy minimization, a heating step (up to 300 K) and a 20 ns equilibration in the NPT ensemble at 1 atm and 300 K. Production simulations were run in the NVT ensemble for at least 1 μs. DNA was described by the parmbsc0/χOL3[5, 6] modification of the Cornell ff99 force field[7]. Water was included by considering the TIP3P model[8], and the Joung-Cheatham parameters for ions were considered[9]. Periodic boundary conditions were implemented, with the Particle Mesh Ewald (PME) accounting for long-range electrostatics and van der Waals interactions truncated at the real space cutoff (9 Å). The SHAKE algorithm was implemented to constrain bonds involving hydrogen atoms, to allow for an integration step of 2 fs.

S2.2 Parameterization procedure

A Boltzmann Inversion was performed by considering the average and fluctuations size of the various bonded interactions for the all-atom simulations performed at 1 pN for the Training Sequences. More in detail, the values of r_0, θ_0 and ϕ_0 were fixed as the averages obtained from the coarse-grained trajectory of atomistic simulations (in the case of ϕ_0, the average was shifted by π since the minimum of the dihedral potential is found at $\phi_0 + \pi$). The elastic constants were tuned in order to match the size of fluctuations computed from the trajectories to the ensemble averages calculated from Equations (1), (2) and (3) in the main text, which we report here for convenience:

$$U_{\rm bond}(r) = k_{\rm bond}(r - r_0)^2 \,, \tag{1}$$

$$U_{\rm angle}(\theta) = k_{\rm angle}(\theta - \theta_0)^2 \,, \tag{2}$$

$$U_{\rm dihedral}(\phi) = k_{\rm dihedral}\left[1 + \cos(\phi - \phi_0)\right] \,. \tag{3}$$

In the case of bonds, the fluctuation size σ_{b} was calculated as

$$\sigma_{\mathrm{b}} = \sqrt{\frac{\int_0^\infty (r - r_0)^2 \cdot r^2 e^{-\frac{U_{\mathrm{bond}}(r)}{k_B T}} dr}{\int_0^\infty r^2 e^{-\frac{U_{\mathrm{bond}}(r)}{k_B T}} dr}}, \tag{S1}$$

with the weight factor r^2 being introduced to account for the three-dimensional nature of the system. Analogously, one has

$$\sigma_{\mathrm{a}} = \sqrt{\frac{\int_0^\pi (\theta - \theta_0)^2 \cdot \sin\theta \, e^{-\frac{U_{angle}(\theta)}{k_B T}} d\theta}{\int_0^\pi \sin\theta \, e^{-\frac{U_{angle}(\theta)}{k_B T}} d\theta}} \tag{S2}$$

for the angles, while

$$\sigma_{\mathrm{d}} = \sqrt{\frac{\int_0^{2\pi} (\phi - \phi_0 - \pi)^2 \cdot e^{-\frac{U_{dihedral}(\phi)}{k_B T}} d\phi}{\int_0^{2\pi} e^{-\frac{U_{dihedral}(\phi)}{k_B T}} d\phi}} \tag{S3}$$

holds in the case of dihedrals. As mentioned above, for each bonded interaction the mean and fluctuation size were computed from the trajectories obtained by coarse-graining the atomistic simulations and the equations relevant to the case at hand were employed to determine the force-field parameters. For instance, for a bond, Equation (S1) was inverted to determine the value of k_{bond} from the knowledge of σ_{b}. This approach neglects the possible effects of the pulling force present in the atomistic simulations. In order to minimize this perturbation, the inversion procedure was performed considering the atomistic trajectories obtained for the smallest pulling force, $f = 1$ pN. It is worth noting that the present approach is fully justified only if the various observables are distributed normally. Among the 202 bonded interactions present in the model, in 85% of cases we found that the histograms are reasonably described by a Gaussian function. In 12% of cases a shoulder was visible, while in a few cases the distribution showed the presence of a secondary peak (e.g. for dihedral 5'-PSPS-3' and step GC). This suggests the presence of two competing conformations for these special instances, which at the current stage was not further addressed in the construction of MADna.

With the force field obtained from Boltzmann Inversion, we performed coarse-grained pulling simulations and compared the obtained elastic constants with the atomistic results. It is evident from Fig.S1 that, while the coarse-grained model gives reasonable values, there is room for improvement. In order to obtain a better agreement, we thus proceeded to a refinement of the bonded parameters. The main goal of this second step was to reproduce the elastic constants of the Training Sequences. In practice, this was done by iteratively adapting the parameters and running coarse-grained simulations of the Training Sequences, from which the elastic constants were computed. Due to the large number of bonded parameters, an exhaustive search of the

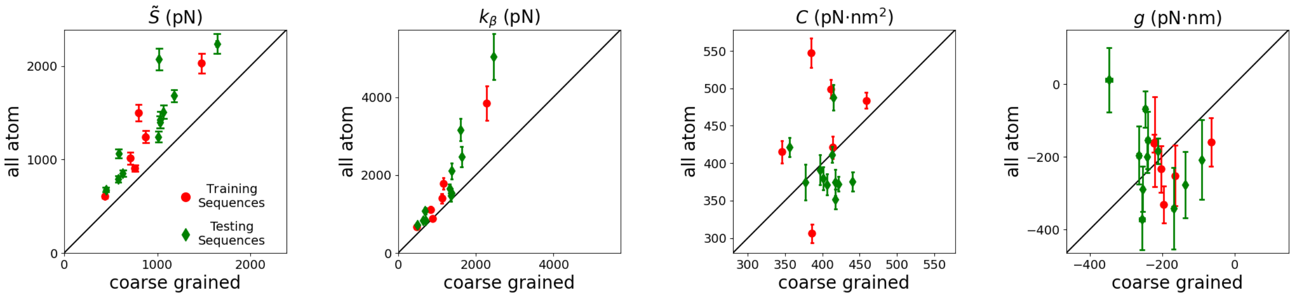

Figure S1: Scatter plot comparing atomistic and coarse-grained Boltzmann-Inversion results for the effective stretching modulus $\tilde{S}$, the crookedness rigidity k_β, the torsion modulus C and the twist-stretch coupling constant g. Training and Testing Sequences are denoted by red circles and green diamonds, respectively. The black line indicates the bisector of the first and third quadrant.

optimal set is beyond current computational possibilities. As discussed in the main text, we adjusted a minimal subset of key parameters which were observed to affect the elastic properties, namely the rigidities of bond 5'-BB-3', angle 5'-PSB-3' and dihedral 5'-SPSP-3'. In order to give an idea of the extent of the tuning of parameters, we mention that the most extreme changes were given by a factor equal to 0.25 (for angle 5'-PSB-3' and step AG) and a 3-fold increase (for dihedral 5'-SPSP-3' and step CG). The optimized set of parameters is reported in Tables S2, S3 and S4 for bonds, angles and dihedrals, respectively.

Bonds

Step	5'-SP-3' r_0 (Å)	5'-SP-3' k_{bond} $\left(\frac{\text{kcal}}{\text{mol Å}^2}\right)$	5'-PS-3' r_0 (Å)	5'-PS-3' k_{bond} $\left(\frac{\text{kcal}}{\text{mol Å}^2}\right)$	5'-BB-3' r_0 (Å)	5'-BB-3' k_{bond} $\left(\frac{\text{kcal}}{\text{mol Å}^2}\right)$
AA	3.74	54.42	4.08	14.59	3.87	5.86
AC	3.74	58.05	4.07	15.03	3.73	8.02
AG	3.75	65.28	4.09	11.51	4.12	3.48
AT	3.75	67.15	4.12	15.87	3.70	18.57
CA	3.76	58.90	4.13	14.82	4.24	5.84
CC	3.77	79.36	4.17	19.01	4.19	2.28
CG	3.76	60.94	4.09	10.43	4.26	4.46
CT	3.75	75.03	4.12	18.69	3.95	4.00
GA	3.74	49.47	4.11	15.13	3.82	6.07
GC	3.71	41.33	4.04	19.21	3.71	9.52
GG	3.76	75.12	4.17	16.75	4.15	3.08
GT	3.74	54.90	4.11	17.11	3.69	13.19
TA	3.76	69.77	4.14	19.27	4.36	8.33
TC	3.76	73.87	4.17	19.81	4.19	2.96
TG	3.76	57.77	4.13	16.39	4.48	3.96
TT	3.76	79.95	4.14	23.67	4.02	4.66

Base	SB r_0 (Å)	SB k_{bond} $\left(\frac{\text{kcal}}{\text{mol Å}^2}\right)$
A	4.89	39.23
C	4.39	44.51
G	5.01	49.13
T	4.46	45.25

WC Pair	BB-WC r_0 (Å)	BB-WC k_{bond} $\left(\frac{\text{kcal}}{\text{mol Å}^2}\right)$
AT	6.09	21.48
CG	5.70	35.95

Table S2: Optimal parameter values for bonds (compare Equation (2)). Units are directly following the convention used in LAMMPS [10], when the option "units real" is selected.

Angles

Step	5'-SPS-3' θ_0 (deg)	5'-SPS-3' k_{angle} $\left(\frac{\text{kcal}}{\text{mol rad}^2}\right)$	3'-PSB-5' θ_0 (deg)	3'-PSB-5' k_{angle} $\left(\frac{\text{kcal}}{\text{mol rad}^2}\right)$	5'-PSB-3' θ_0 (deg)	5'-PSB-3' k_{angle} $\left(\frac{\text{kcal}}{\text{mol rad}^2}\right)$
AA	94.12	26.15	115.33	32.47	113.21	6.21
AC	91.81	27.73	116.09	40.51	109.29	20.74
AG	94.11	23.84	115.28	35.91	119.24	2.44
AT	92.83	33.04	114.73	43.58	105.79	20.24
CA	96.24	22.69	119.22	38.06	110.49	13.46
CC	94.52	32.47	117.56	51.38	111.33	19.19
CG	95.46	21.80	120.50	37.77	113.34	8.21
CT	92.87	38.18	117.21	51.27	106.69	4.26
GA	95.14	22.91	111.77	29.27	108.34	1.56
GC	92.66	25.46	111.94	33.19	106.68	10.49
GG	94.25	34.28	109.64	52.70	121.94	11.45
GT	94.46	28.77	111.28	37.18	104.88	26.48
TA	94.39	26.40	119.62	40.82	114.26	8.79
TC	93.81	34.18	121.01	48.59	107.49	3.25
TG	93.12	29.86	120.09	42.72	118.63	15.01
TT	92.66	46.62	119.89	57.92	104.95	13.15

WC Pair	SBB θ_0 (deg)	SBB k_{angle} $\left(\frac{\text{kcal}}{\text{mol rad}^2}\right)$
AT	154.31	51.58
CG	138.35	53.86
GC	158.75	52.15
TA	132.80	49.68

Table S3: Optimal parameter values for angles (compare Equation (3)). Note that, following the convention in LAMMPS [10], θ_0 is expressed in degrees while k_{angle} has units of kcal/mol rad^2. Units are directly following the convention used in LAMMPS, when the option "units real" is selected. In the case of SBB, the Watson-Crick pairs are ordered by listing first the base bonded to the sugar moiety.

Dihedrals

Step	5'-SPSP-3' ϕ_0 (deg)	5'-SPSP-3' k_{dihedral} $\left(\frac{\mathrm{kcal}}{\mathrm{mol}}\right)$	5'-PSPS-3' ϕ_0 (deg)	5'-PSPS-3' k_{dihedral} $\left(\frac{\mathrm{kcal}}{\mathrm{mol}}\right)$	5'-SPSB-3' ϕ_0 (deg)	5'-SPSB-3' k_{dihedral} $\left(\frac{\mathrm{kcal}}{\mathrm{mol}}\right)$
AA	0.43	59.55	22.36	4.72	-142.34	13.82
AC	-1.50	23.66	27.39	4.82	-141.29	19.09
AG	-2.16	47.34	26.73	5.56	-145.79	10.12
AT	-0.87	19.10	24.42	7.50	-137.42	27.14
CA	-2.64	25.63	20.00	5.32	-144.63	10.96
CC	-3.69	44.82	23.61	11.51	-140.36	21.61
CG	-0.79	54.62	26.55	4.11	-149.56	7.56
CT	-0.50	57.43	24.68	10.46	-136.72	27.53
GA	-0.47	50.07	19.19	4.60	-139.81	16.59
GC	0.98	52.40	17.71	2.75	-137.94	21.41
GG	-3.17	42.78	23.55	10.89	-141.02	13.63
GT	0.70	25.74	19.03	5.61	-135.20	28.05
TA	-2.86	19.63	22.11	8.35	-143.86	14.21
TC	0.07	60.41	19.32	10.76	-138.01	23.55
TG	-2.67	32.65	26.50	7.75	-146.92	9.02
TT	2.02	77.55	20.52	15.66	-135.61	34.02

Step	3'-SPSB-5' ϕ_0 (deg)	3'-SPSB-5' k_{dihedral} $\left(\frac{\mathrm{kcal}}{\mathrm{mol}}\right)$	5'-PSBB-3' ϕ_0 (deg)	5'-PSBB-3' k_{dihedral} $\left(\frac{\mathrm{kcal}}{\mathrm{mol}}\right)$	3'-PSBB-5' ϕ_0 (deg)	3'-PSBB-5' k_{dihedral} $\left(\frac{\mathrm{kcal}}{\mathrm{mol}}\right)$
AA	163.97	10.39	20.94	10.50	-124.78	10.12
AC	166.22	10.91	19.79	13.64	-120.21	7.45
AG	165.09	8.94	22.43	6.70	-120.17	8.56
AT	164.45	14.86	21.44	23.90	-116.14	8.18
CA	155.62	6.91	23.13	7.62	-117.01	8.98
CC	157.37	13.92	8.61	17.91	-126.38	13.92
CG	159.63	5.56	38.23	6.39	-113.08	8.85
CT	158.95	13.43	21.42	18.04	-119.92	10.04
GA	165.40	11.53	21.21	10.67	-124.79	7.92
GC	165.80	11.04	23.86	15.31	-114.10	6.71
GG	166.73	15.04	10.35	7.24	-130.58	7.91
GT	165.12	14.54	22.28	24.12	-113.48	7.56
TA	152.13	8.69	27.76	8.40	-112.76	12.11
TC	149.37	12.01	19.82	12.94	-112.15	12.99
TG	154.62	6.71	35.89	6.90	-110.67	10.70
TT	151.88	15.72	21.43	21.30	-115.81	14.63

Table S4: Optimal parameter values for dihedrals (compare Equation (4)). Units are directly following the convention used in LAMMPS [10], when the option "units real" is selected.

S3 List of sequences

S3.1 Model definition and benchmark simulations

Training Sequences

In order to determine the parameters of the bonded interactions, a matching procedure was employed to reproduce the results of a set of atomistic simulations exhaustively accounting for all the possible base steps [2]. This set is here referred to as “Training Sequences” and is composed of the six molecules polyAA, polyAC, polyAG, polyAT, polyCG and polyGG, each consisting of a pentamer of dinucleotides flanked by two CGCG handles, introduced to avoid end effects. Hence, the sequences are (handles are evidenced by the presence of a dash in the sequence):

polyAA ↔ 5’-CGCG-AAAAAAAAAA-CGCG-3’,

polyAC ↔ 5’-CGCG-ACACACACAC-CGCG-3’,

polyAG ↔ 5’-CGCG-AGAGAGAGAG-CGCG-3’,

polyAT ↔ 5’-CGCG-ATATATATAT-CGCG-3’,

polyCG ↔ 5’-CGCG-CGCGCGCGCG-CGCG-3’,

polyGG ↔ 5’-CGCG-GGGGGGGGGG-CGCG-3’.

Testing Sequences

The predictive power of the model was tested by comparing the coarse-grained and atomistic results for a second set of sequences [2, 3], which are referred to as “Testing Sequences”. Some of these sequences have direct biological relevance, such as the DNA Unwinding Element (DUE), a TATA-box element (TATA) and a Transcription Factor Binding Site (TFBS). As before, the handles are evidenced by the presence of a dash in each sequence.

DDD ↔ 5’-CGCG-CGCGAATTCGCG-CGCG-3’,

TATA ↔ 5’-CGCG-TATAAAAG-CGCG-3’,

TFBS ↔ 5’-CGCG-GGATGGGAG-CGCG-3’,

G4CG4 ↔ 5’-CGCG-GGGGCGGGG-CGCG-3’,

G4AAG4 ↔ 5’-CGCG-GGGGAAGGGG-CGCG-3’,

DUE ↔ 5’-CGCG-GATCTATTTATTT-CGCG-3’,

A4TA4 ↔ 5'-CGCG-AAAATAAAA-CGCG-3',

A4GGA4 ↔ 5'-CGCG-AAAAGGAAAA-CGCG-3',

A8T ↔ 5'-CGCG-AAAAAAAAT-CGCG-3',

A8GG ↔ 5'-CGCG-AAAAAAAAGG-CGCG-3',

All-steps ↔ 5'-GCG-CAATGGAGTA-CGC-3'.

S3.2 Persistence-length simulations

Sequence-averaged

P1 ↔ 5'-ACTCATCGAC TGTATTGTGC TCGGTAGATT GAAGGAGTAG CTTAGTCCTC ATAAAGCGGA CGAACATCGT TGCTAGAAAA TCCCTGGAAT GCACGTAGAT-3'

P2 ↔ 5'-GCAACAAGGT ATCAAGTTAT GCAGGGTCTT TCTGGGTTGT CGTACTAGCT GGATTAAATG CGAAGAGTCG CTAAAACCAC ACTACCAAAC ACTAGTAATA-3'

P3 ↔ 5'-CTAGACATTA TGGTTGGTGA GACCCGAGCC TTCTCTAAGA GACAACGCTA ATGTCTCGGA CGGTAGTCCG TCCCCTGAAT ATGCAATACA CCGTCTCTGA-3'

P4 ↔ 5'-GTCGTTTTCA CTCCCAGACC CATATGTTAT GTATTGTCCC AGTAATGTGT TGGTGCTAGA GCTAGCCTAT GTTCCAACCA CCAGCCTTCT GACTTTACAA-3'

P5 ↔ 5'-ACTTCACTTG TCGAAGTATT ACCTCATTAG AATAAATATT TTTATTCCAA GTCTCATCAT GGGGAAGGTG CTCTCATCTC TTTGCTGCGT AGTTTTATGG-3'

P6 ↔ 5'-GTTTGGTGCT TAGACCCGAT GCAACGGGCA TCATTACCCG GGTCCCCAAG GCCTAAGTAT GAATGTGTTA GACCCCCATC TTAGAACTGC TGGCGGCAAT-3'

P7 ↔ 5'-ATCGCGGGTT TAGGCTCGTC TCATTAGTTA AATTATGTGC GTCATAGAGA AGGTTTGAGC CTCGCATGAA GCTGTCAACG CCGATGTAGT TGTGTACGTG-3'

P8 ↔ 5'-GACTAACTAA GCACTGGGGA GTAACTGCTC CAGACTGTTA GACCTCTCAC TCCTAAAGGT ATTGAGAAAG CGTACTTCTA CATTCATTTG CACTGGTATC-3'

P9 ↔ 5'-TACCCGATAC GCTATCAGAA AGCATGGGCT AGTAAGCACA ATGGTACTGA GTACTCTACG TACGCAACCG TCCTTATTTA AATGCGCGGC CAAAACCAAA-3'

P10 ↔ 5'-CCAGGCGCCC GACTGCGATG GGCGATGCAA CTCCGAGTAG TTTAATTCGA GGGGAGCTGG AGGCATTGTA TCAGATTTTG AGCGGTGGCT TTTACCAGAG-3'

P11 ↔ 5'-GAGCATGCCG ATGTCCTGGC GACACGAAAC CCCCGAGCGC AACGCTGCTC ACAATGTTTT AGTATGATCC GACGTATGTA AAACGTCGTT GGGACCATTG-3'

P12 ↔ 5'-CACTTCAGGC GTCCTCTCCG CGGAACACTA GTACTCCCGT TCGGATGCTG GGGCTCCTTC TGTGAGGATT CAAAATGGGA AAGATCCCAA CCGGTGTGCC-3'

P13 ↔ 5'-TATAGGTCTG CGGCTCCCTT CTGTCAACTT GTCCTGCCAT CTCCCAAGTC GCTACGTGAG TTAGTCCCTT TAGCTGGAGC GAGGCATCCC TACAAGCCTG-3'

P14 ↔ 5'-ACGTATCGTG ACGAGATAGT CCTACTACTA GGAAGAAATC CCGGCTAGGT ATAAGAGGCC TGACCTAAAT CAAGAACAGA TGGGTTCAAT GAACTCATAG-3'

P15 ↔ 5'-CAAACCGCCT TGGGCTCGAG CCTTCGGGCG TGACGGGACG AGTCAATTTC AGTCCATACG CTAGTTATGG TATACAGGCG TCGGAGTTGC CCTTGATGGA-3'

P16 ↔ 5'-GGTTGCGATG AAAAGCTAGT GGTATTTGAC AAGCAGGCTT CAGTCACCCT GCGGCGTAGT TTTGTTAGAC ACTTCCCGTC TAGCTGTCGT ACATTTACGT-3'

P17 ↔ 5'-GTCTTTCGGG GGACGATAGG CGATCCCAAC TCTCTCTATC TAAGACGAAA TCAGTGCAAA AGTAATAGGG ATAACGTCCG CGTGTGTCAT TCGCCGTATC-3'

P18 ↔ 5'-GCCAGGCGGC GGTACATGGA GGATGCAAGA GTCTTATTGT GTTGTATAAG GCTTCACAGG TACCTATGCA TAGGCGGCTA AACTCTCACC CATGCCGACA-3'

P19 ↔ 5'-TTGAGAGATC GCCACACACT CAAGAGAGTG GACTCATGAA ACTTCCGACG TACGGACGCC GGATGCGGGG TATTATGGGC TGTAGCCGTA TGCGTGCCGC-3'

P20 ↔ 5'-CCGATCACAT GTCCGGCGCA ATCGGTATTT TGTGGGTCTA CAGGTACTAT GTTATTCGTA CGTTCTGGCC CCGATCACGA ACCGATGAGC ACAGATGCTG-3'

Sequence-dependent

ACAT ↔ 5'-TACACATATA TACACATACA TACACACATA CATATATATA CACACACATA TACATACATA TACATACATA CATATATACA CACATACATA CACATATATA-3'

ACCAGG ↔ 5'-AGGACCACCA GGAGGACCAG GAGGAGGACC ACCACCAGGA CCACCAGGAC CAGGAGGAGG ACCACCAGGA CCACCAGGAG GAGGACCACC AGGACCAGGA-3'

ACGAGC ↔ 5'-CACGACGACG AGCACGAGCA GCACGACGAG CACGAGCAGC AGCACGAGCA CGACGAGCAG CACGACGAGC AGCACGACGA GCACGACGAC GAGCAGCAGC-3'

AGAT ↔ 5'-AGAGATATAT AGAGATAGAT AGAGAGATAG ATATATATAG AGAGAGATAT AGATAGATAT AGATAGATAG ATATATAGAG AGATAGATAG AGATATATAT-3'

AGC ↔ 5'-GCAGCTAGCA GCAGCAGCAG CAAGCAGCAG CAGCAGCGAG CAGCAGCAGC AGCTAGCAGC AGCAGCAGCA AGCAGCAGCA GCAGCGAGCA GCAGCAGCAG-3'

CAA ↔ 5'-CAACAACAAG AACAACAACA ACAACAACCA ACAACAACAA CAACAACCAA CAACAACAAG CAACAACAAC AACAACAACC AACAACAACA ACCAACAACA-3'

CAACTT ↔ 5'-CTTCAACAAC TTCAACTTCT TCAACAACAA CTTCTTCTTC AACAACTTC[illegible] ACAACTTCTT CAACTTCAAC AACTTCTTCA ACTTCAACTT CTTCAACAAC-3'

CAGT ↔ 5'-AGCAGTCAGT CAGTCAGTCA GTCAGTCAGT CTGACAGTCA GTCAGTCAGT CAGTCAGTCA GTCAGTCTGA CAGTCAGTCA GTCAGTCAGT CAGTCAGTCA-3'

CATCTA ↔ 5'-ATCTACTACA TCTACTACTA CATCATCATC TACATCTACT ACATCATCTA CTACATCATC TACTACATCA TCTACATCTA CATCTACTAC ATCATCTACT-3'

HPL1 ↔ 5'-AGCGATTCGG CATTCGATTC GCGCATTCGA TTCGCATTCG ATTCGGCATT CGATTCGGCA TTCGATTCGC ATTCGATTCA TTCATTCGAT TCGGCATTCG-3'

HPL2 ↔ 5'-ACGACGAACG ACGAACGACG ACGAACGAAC GACGACGAAC GACGAACGAC GACGAACGAC GACGAACGAC GAGACGAACG AACGACGACG AACGACGAAC-3'

LPL1 ↔ 5'-GCAGTAGTAG CCTAGTAGCC TAGTAGCCTA GTAGCCTAGT AGCCTAGTAG CCTAGTAGCC TAGTAGCCTA GTAGCCTAGT AGCCTAGTAG CCTAGTAGCC-3'

LPL2 ↔ 5'-AGGGCCATAG GCATGCATAG GCATAGGCCA TGGCATAGGC ATTAGGCATG CATAGGCATA GGCATGGCAT AGGCATTAGG CATGCATAGG CATAGGCATG-3'

SG1 ↔ 5'-AGCAGCTAGC TAGCGCGATG CCCAGCTGAG ATCGACGATC GATGGCGATT ATCAGCTAGC AGCTAGCGAT CGACGCGCGA TGCGCAGCTG AGCTAGCTGA-3'

S3.3 Stretch-torsion simulations

Sequence-averaged

ST1 ↔ 5'-GATTAATGAC GGACAACTCT GCTGTCCTGA CGCGGCGAAA-3'

ST2 ↔ 5'-CGAATGGACG TGGAGGTAGT CCGATTTCGA TCCAAGGTGA-3'

ST3 ↔ 5'-TTCTCCTGCA ACTGGATCCC GCTTGATGTT TTAGGTTAAC-3'

ST4 ↔ 5'-TTCTAACTGA TTTGGCTAGC GTATACTTCT GTATCTGTCC-3'

ST5 ↔ 5'-CCTCACATTT TAGCACCGCC TCCCGATAAC TTAAGGGTTC-3'

ST1-short ↔ 5'-GGACAACTCT GCTGTCCTGA-3'

ST2-short ↔ 5'-TGGAGGTAGT CCGATTTCGA-3'

ST3-short ↔ 5'-ACTGGATCCC GCTTGATGTT-3'

ST4-short ↔ 5'-TTTGGCTAGC GTATACTTCT-3'

ST5-short ↔ 5'-TAGCACCGCC TCCCGATAAC-3'

Phased A-tracts

A-tract-1 ↔ 5'-CCCCAAAAAT TTTCAAATTT TTTCTCGAAA AATTGCAAAA-3'

A-tract-2 ↔ 5'-CAAATAATCA TTCAAAAAAA AAAACTTTCT AAAAAATCTC-3'

A-tract-3 ↔ 5'-CAAAAATTTT CAAATTTTTT CTCGAAAAAT TGCAAAAAAT-3'

A-tract-4 ↔ 5'-GAAAAAAAAT GCTTAAAATT TCAGAAAATG TTAAAAATTC-3'

A-tract-5 ↔ 5'-AAAAATTCCG AAAATTGGTT AAAAAATTTT TTTAAAAAAC-3'

S3.4 Twist-bend simulations

TB1 ↔ 5'-ACAGAACTAG TCGCTACGTA CATGGTCCAT CGCTGGTATA TGCATAATAT AGTGTGAGAA ACCCAATGGG CTATGCGCTC TTTGGGCCAC AGCCCCTGCA AAAATACTTG TGTACTACCT TCTCCTCAGC TGTCAGAGAA TACATGATCG-3'

TB2 ↔ 5'-TTCTAGGAGA CATGTATATC GGATATGTCA TTGTATTAGC GAAGAGCAGG AGGAAACGCT GCTCCTGTAA CAGGGTTGGT TCGGTGCAGA GGGGTAATTT GGACTCCAGC CCTACTGTCA AGTAACTAGG TTGCACAGAC CATTGAGAAC-3'

TB3 ↔ 5'-GTAAAGCGAT AGCAAGATGA CCTATAGTGA TCTTAAACGT CCACAGAGAT CAGTTTTTCC TCTCATATTC AGGGTCCGTA GAGATACGGG AGGGGATTAC TCAACAGT[illegible] GTGGGGGGGA TATATAACTC TCTTCTTGTC CGGTCGATGA-3'

S4 Determination of helical parameters

S4.1 Best fitting cylinder

The helical radius and axis were defined by considering the cylinder which best fitted the positions of the phosphates on the two strands (see Fig.4 in the main text). The fit was performed by implementing the algorithm proposed in Ref.[12], where the optimal axis is found by iteratively proposing random orientations and selecting the one minimizing the square error. For the selected axis, the radius is obtained as a byproduct of the computation.

Here we report for convenience the main steps of the derivation in Ref.[12]. An infinite cylinder is identified by a unit-length axis $\hat{\boldsymbol{H}} \equiv (h_0, h_1, h_2)$, a radius R and a center $\boldsymbol{C}$ chosen arbitrarily along the axis. By introducing the identity matrix I and the transposed vector $\hat{\boldsymbol{H}}^T$, the rejection operator $P \equiv I - \hat{\boldsymbol{H}}\hat{\boldsymbol{H}}^T$ projects any vector onto the plane perpendicular to $\hat{\boldsymbol{H}}$. By construction, one has $P^T = P$ and $P^2 = P$. For any point $\boldsymbol{X}$, the quantity $(\boldsymbol{X} - \boldsymbol{C})^T P^T P (\boldsymbol{X} - \boldsymbol{C}) = (\boldsymbol{X} - \boldsymbol{C})^T P (\boldsymbol{X} - \boldsymbol{C})$ corresponds to the square of the distance separating $\boldsymbol{X}$ from the axis. Hence, if $\boldsymbol{X}$ lies on the surface of the cylinder, the following formula holds:

$$(\boldsymbol{X} - \boldsymbol{C})^T P (\boldsymbol{X} - \boldsymbol{C}) = R^2 . \tag{S4}$$

For fitting purposes, the error function to be minimized is thus

$$E\left(R^2, \boldsymbol{C}, \hat{\boldsymbol{H}}\right) \equiv \sum_{i=1}^{n} \left[(\boldsymbol{X}_i - \boldsymbol{C})^T P (\boldsymbol{X}_i - \boldsymbol{C}) - R^2\right]^2 , \tag{S5}$$

where $\boldsymbol{X}_i, i = 1, \dots, n$ are the points to be fitted. The best-fitting cylinder is found by minimizing the error function with respect to the radius, center and axis of the cylinder. Denoting as R_i the distance separating $\boldsymbol{X}_i$ from the axis, i.e. $R_i^2 = (\boldsymbol{C} - \boldsymbol{X}_i)^T P (\boldsymbol{C} - \boldsymbol{X}_i)$, the condition $\partial E / \partial R^2 = 0$ implies

$$R^2 = \frac{1}{n} \sum_{i=1}^{n} R_i^2 . \tag{S6}$$

The condition $\partial E / \partial \boldsymbol{C} = 0$ provides an equation which does not uniquely identify $\boldsymbol{C}$, since any point along the axis is an acceptable solution. Choosing for the center the point satisfying the supplementary condition $P\boldsymbol{C} = \boldsymbol{C}$, it can be shown [12] that

$$\boldsymbol{C} = \frac{\bar{A}}{\text{Trace}\left(\bar{A}A\right)} \left[\frac{1}{n} \sum_{i=1}^{n} \left(\boldsymbol{X}_i^T P \boldsymbol{X}_i\right) \boldsymbol{X}_i\right] , \tag{S7}$$

where

$$A \equiv P \left(\frac{1}{n} \sum_{i=1}^{n} \boldsymbol{X}_i \boldsymbol{X}_i^T\right) P \tag{S8}$$

and

$$\bar{A} \equiv S A S^T \,, \tag{S9}$$

with S being the skew symmetric matrix

$$S = \begin{pmatrix} 0 & -h_2 & h_1 \\ h_2 & 0 & -h_0 \\ -h_1 & h_0 & 0 \end{pmatrix} \,. \tag{S10}$$

The equation stemming from the condition $\partial E / \partial \hat{\boldsymbol{H}} = 0$ cannot be solved analytically, thus preventing the possibility of building a closed-form solution for the direction of the optimal cylinder. Nevertheless, for any given axis $\hat{\boldsymbol{H}}$, the optimal radius and center are readily found by means of Equations (S6) and (S7). Substitution of these values into Equation (S5) leads to a conditional least-square error $E_{\hat{\boldsymbol{H}}}$, i.e. the minimum error associated to the chosen axis $\hat{\boldsymbol{H}}$. Hence, the fit can be performed by considering many randomly-oriented trial axes and selecting the one with the lowest value of $E_{\hat{\boldsymbol{H}}}$.

In the present case, 10^4 iterations were considered to search for the optimal cylindrical direction. We have counterchecked numerically the efficacy of this approach by extracting coordinate points from artificially-created double helices with known axes and radii, chosen at random within the typical ranges found for DNA. More in detail, we built 10^4 double helices with orientation $\hat{\boldsymbol{h}}$ chosen at random. The radius r and periodicity l were also chosen at random within the intervals $[0.5\text{ nm}, 5\text{ nm}]$ and $[1\text{ nm}, 10\text{ nm}]$, respectively, which largely encompass the ranges of values typical of double-stranded DNA. The two helices were shifted in order to form a minor and major groove with sizes following the ratio 4:7, as found in the crystal structure of the Drew-Dickerson dodecamer [13]. For each helix, twenty equally-spaced points were extracted within two periodicities, mimicking the positions of the phosphate groups along DNA fragments of length comparable to the Training and Testing Sequences considered in this work. For each helix, the search for the optimal axis was run by considering 10^4 random orientations. The radius R and axis $\hat{\boldsymbol{H}}$ of the fitted cylinder were then compared to r and $\hat{\boldsymbol{h}}$. As for the radii, we considered the relative error $e_R \equiv |R - r|/r$. On average, it was found that $\langle e_R \rangle \simeq 0.002$. Moreover, its maximum value was 0.05 and an error $e_R > 0.01$ was found in only about 1% of the simulated helices. As for the orientation, we computed the angle θ formed by $\hat{\boldsymbol{h}}$ and $\hat{\boldsymbol{H}}$, i.e. $\cos\theta \equiv \hat{\boldsymbol{h}} \cdot \hat{\boldsymbol{H}}$. On average, we found that $\langle \theta \rangle \simeq 0.7$ degrees, with a maximum value of 2.6 degrees and $\theta > 1.5$ degrees found in about 4% of cases. Overall, these results corroborate the robustness of the algorithm for the determination of the helical features of double-stranded DNA.

S4.2 Comparison between computed h-twist and 3DNA definition

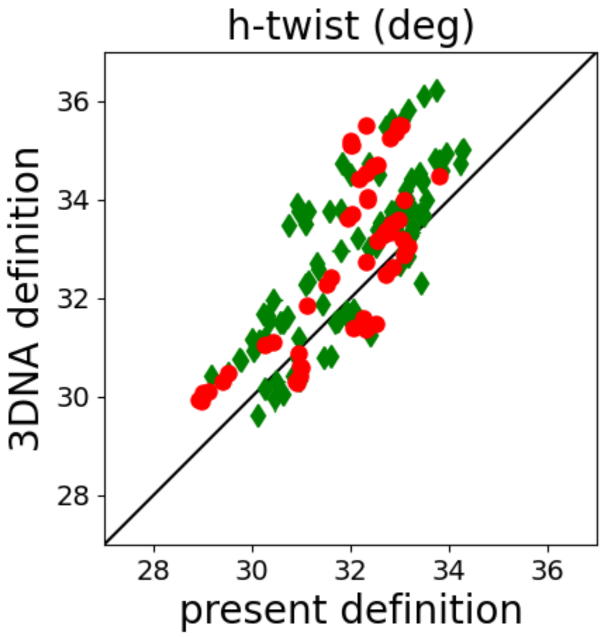

Figure S2: Scatter plot comparing the h-twist computed for the atomistic trajectories according to the present definition and the values obtained by means of 3DNA [14]. Red circles and green diamonds represent steps from Training and Testing Sequences, respectively. The black line depicts the bisector of the first and third quadrant.

S5 Benchmark Simulations

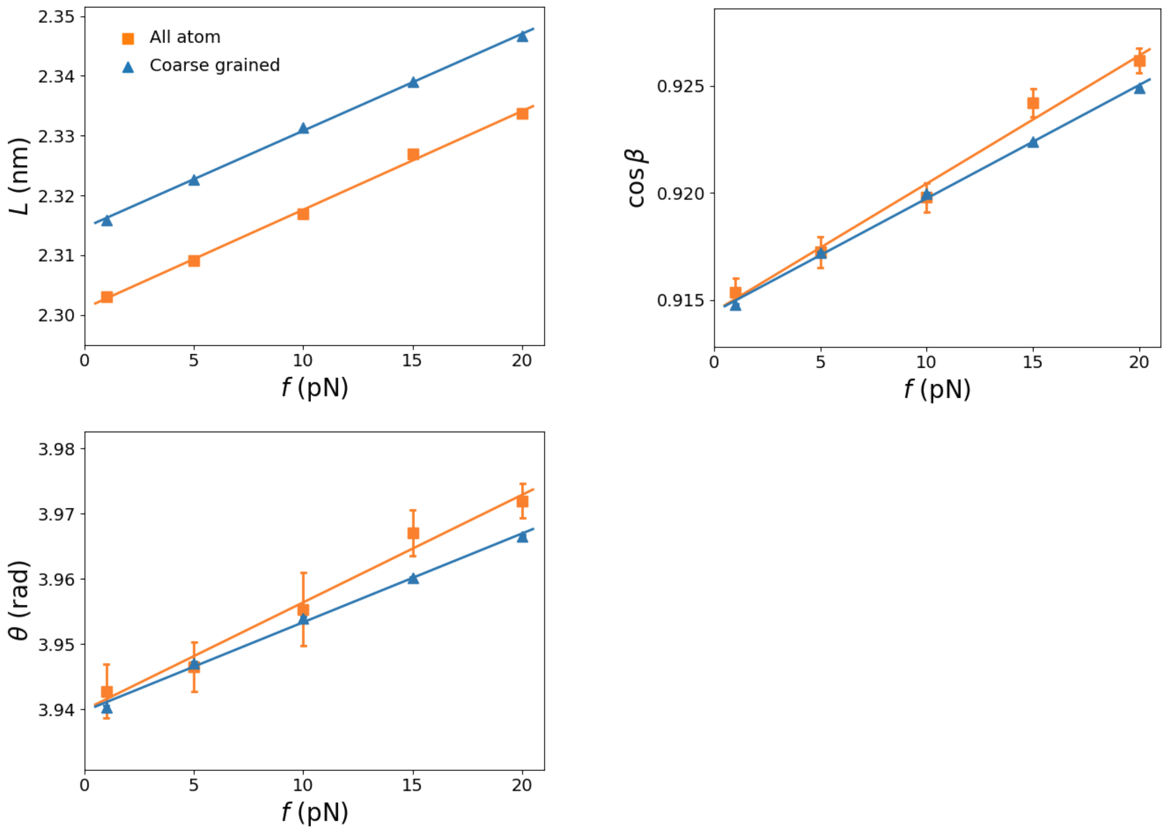

Figure S3: Linear dependence of extension L (top-left panel), cosine of crookedness $\cos\beta$ (top-right panel) and cumulate h-twist θ (bottom-left panel) as a function of the force f for a representative sequence (TATA). All-atom and coarse-grained results are denoted by orange squares and blue triangles, respectively. The lines show the linear fits of the data. There is a systematic overestimation of L in the coarse-grained simulations, which however is very small, as it corresponds to roughly 0.002 nm per base-pair step.

Training Sequences

Name	Simulation Type	$\tilde{S}$ (pN)	k_β (pN)	C (pN·nm^2)	g (pN·nm)
AA	coarse grained	2080 ± 15	3706 ± 37	419 ± 2	-162 ± 8
	all atom	2031 ± 105	3848 ± 444	421 ± 15	-159 ± 124
AC	coarse grained	972 ± 5	1058 ± 6	523 ± 3	-257 ± 6
	all atom	1011 ± 65	1116 ± 73	548 ± 20	-252 ± 83
AG	coarse grained	1163 ± 6	1460 ± 9	289 ± 2	-230 ± 5
	all atom	1240 ± 61	1400 ± 120	306 ± 12	-234 ± 64
AT	coarse grained	899 ± 5	897 ± 5	479 ± 2	-331 ± 6
	all atom	905 ± 35	878 ± 46	499 ± 12	-331 ± 50
CG	coarse grained	1442 ± 10	1859 ± 15	410 ± 2	-159 ± 6
	all atom	1494 ± 87	1785 ± 151	415 ± 15	-159 ± 67
GG	coarse grained	575 ± 2	671 ± 3	492 ± 3	-175 ± 4
	all atom	605 ± 15	675 ± 21	484 ± 11	-164 ± 24

Table S5: Comparison between atomistic and coarse-grained results for the Training Sequences. The elastic constants were computed discarding the handles from the analysis.

Testing Sequences

Name	Simulation Type	$\tilde{S}$ (pN)	k_β (pN)	C (pN·nm^2)	g (pN·nm)
DDD	coarse grained	1785 ± 14	2753 ± 29	372 ± 2	-124 ± 6
	all atom	2070 ± 113	2475 ± 265	422 ± 13	-208 ± 110
TATA	coarse grained	1427 ± 10	1727 ± 12	420 ± 2	-353 ± 9
	all atom	1397 ± 65	1536 ± 113	371 ± 14	-372 ± 83
TFBS	coarse grained	781 ± 5	891 ± 5	388 ± 2	-243 ± 6
	all atom	851 ± 34	832 ± 45	380 ± 15	-289 ± 62
G4CG4	coarse grained	647 ± 4	741 ± 4	466 ± 3	-211 ± 5
	all atom	678 ± 25	726 ± 37	372 ± 10	-184 ± 35
G4AAG4	coarse grained	773 ± 3	933 ± 5	424 ± 2	-216 ± 4
	all atom	791 ± 33	837 ± 45	375 ± 13	-199 ± 45
DUE	coarse grained	1412 ± 10	1740 ± 15	380 ± 2	-217 ± 6
	all atom	1421 ± 91	1466 ± 148	391 ± 20	-342 ± 113
A4TA4	coarse grained	1431 ± 14	1790 ± 15	425 ± 2	-311 ± 9
	all atom	1508 ± 71	2103 ± 197	374 ± 18	-196 ± 80
A4GGA4	coarse grained	1402 ± 8	1967 ± 13	388 ± 2	-215 ± 6
	all atom	1242 ± 57	1648 ± 116	352 ± 13	-155 ± 79
A8T	coarse grained	2303 ± 33	3741 ± 46	427 ± 2	-298 ± 13
	all atom	2235 ± 102	5039 ± 582	488 ± 17	12 ± 89
A8GG	coarse grained	1629 ± 10	2532 ± 20	410 ± 2	-197 ± 7
	all atom	1681 ± 65	3166 ± 285	411 ± 11	-69 ± 50
All-steps	coarse grained	785 ± 3	880 ± 4	396 ± 2	-193 ± 4
	all atom	1064 ± 48	1080 ± 86	374 ± 24	-277 ± 91

Table S6: Comparison between atomistic and coarse-grained results for the Testing Sequences. The elastic constants were computed discarding the handles from the analysis.

S5.1 Analysis of A8T

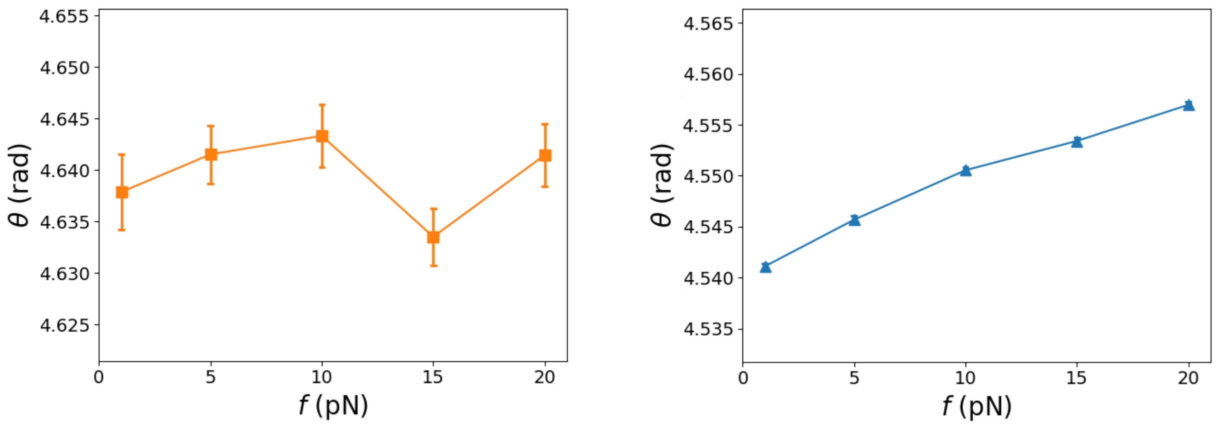

Figure S4: Dependence of cumulate h-twist θ on the force f for sequence A8T. Left and right panel correspond to all-atom and coarse-grained results, respectively.

As mentioned in the main text, in the case of sequence A8T a discrepancy is observed between the coarse-grained and atomistic predictions. The coarse-grained model predicts $g = (-298 \pm 13)$ pN·nm, while $g = (12 \pm 89)$ pN·nm according to atomistic simulations (cfr Table S6). This discrepancy can be ascribed to the noisy response to the pulling force observed in the latter case for large values of f (Fig.S4 left). Indeed, if one limits the analysis to low forces, $f \leq 10$ pN, the atomistic prediction becomes $g = (-242 \pm 237)$ pN·nm, which is in agreement with the coarse-grained prediction, although being characterized by a large error. The flucutating behavior of the cumulate h-twist observed for atomistic simulations is likely due to a lack of full convergence at large forces.

S6 Robustness of persistence-length results with respect to definition of tangent vectors

The estimation of the persistence length of dsDNA may be sensitive to the choice made for the tangent vectors $\hat{\boldsymbol{t}}_i$ [11]. In order to assess the robustness of our results, we considered four different definitions for the tangent vectors, to which we refer as Sugars, Cylinder, Bases and Helical axis definitions.

The Sugars definition corresponds to the one reported in the main text. For the Cylinder definition, we define the displacement vector as $\boldsymbol{R}_{ij} \equiv \boldsymbol{O}_j - \boldsymbol{O}_i$, where $\boldsymbol{O}_i$ is the centre of the cylinder defining the helical axis in the fragment between $i-5$ and $i+5$ (cfr Section S4.1). For the Bases definition $\boldsymbol{O}_i$ is the geometrical center of the base beads, $\boldsymbol{O}_i = (\boldsymbol{B}_{1,i} + \boldsymbol{B}_{2,i})/2$. Finally, for the helical axis definition the local tangent vector $\hat{\boldsymbol{t}}_i$ was defined as the helical axis of the fragment including the base pairs $i-5, \ldots, i+5$

With these definitions, we computed the correlation functions c_p, c_s and c_d according to Equations (12), (13) and (14) in the main text. The resulting functions are plotted in Fig.S5. Extreme values and averages of the corresponding l_p, l_s and l_c are reported in Table S7.

Definition	Statistical Feature	l_p (nm)	l_s (nm)	l_d (nm)
Sugars	Average	56 ± 1	485 ± 42	64 ± 1
	Min	46	199	56
	Max	64	796	77
Cylinder	Average	63 ± 1	1380 ± 327	69 ± 1
	Min	54	361	60
	Max	72	6463	85
Bases	Average	59 ± 1	1201 ± 453	65 ± 1
	Min	51	273	61
	Max	68	9639	73
Helical axis	Average	60 ± 1	1641 ± 346	65 ± 1
	Min	49	254	61
	Max	69	5115	71

Table S7: Average, minimum and maximum values of l_p, l_s and l_d according to the various definitions.

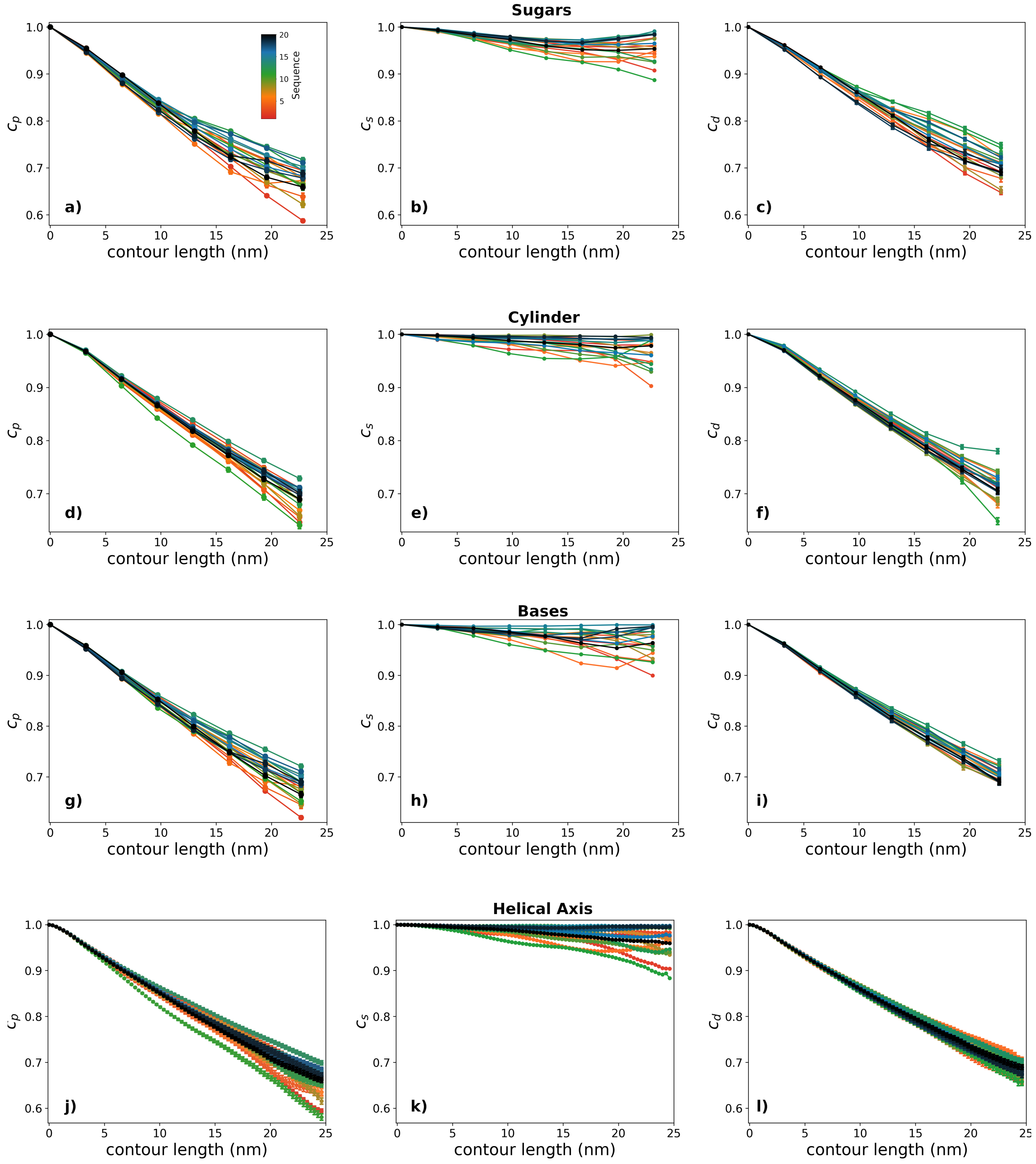

Figure S5: Correlation functions c_p, c_s and c_d obtained for the various sequences according to the four definitions. The sequences are listed in Section S3.2 and are numbered according to the colour code reported in panel a.

S7 Length dependence of elastic constants

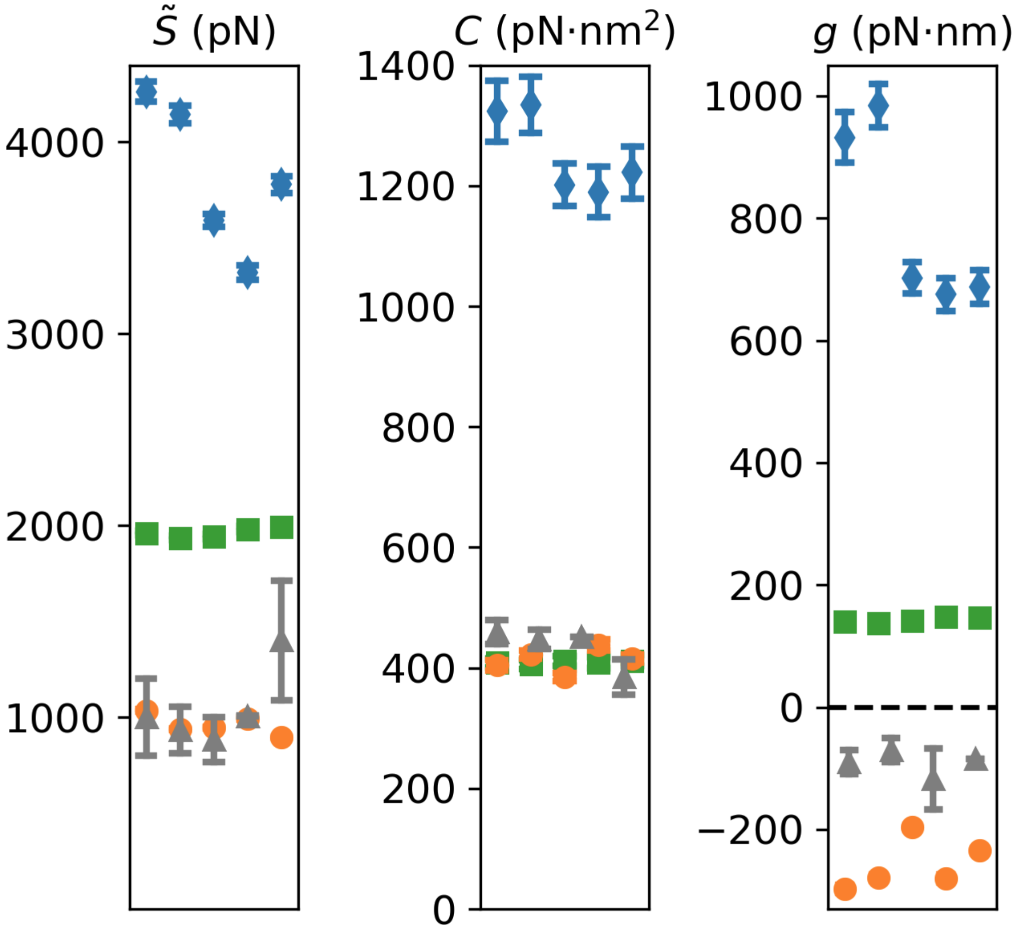

Figure S6: Elastic constants obtained for MADna (orange circles), oxDNA2 (green squares) and 3SPN2C (blue diamonds) for sequences made of twenty base pairs, which are listed as ST1-short,. . . ,ST5-short in Section S3.3.

S8 Sequence-dependent helical pitch and persistence length for oxDNA2 and 3SPN2C

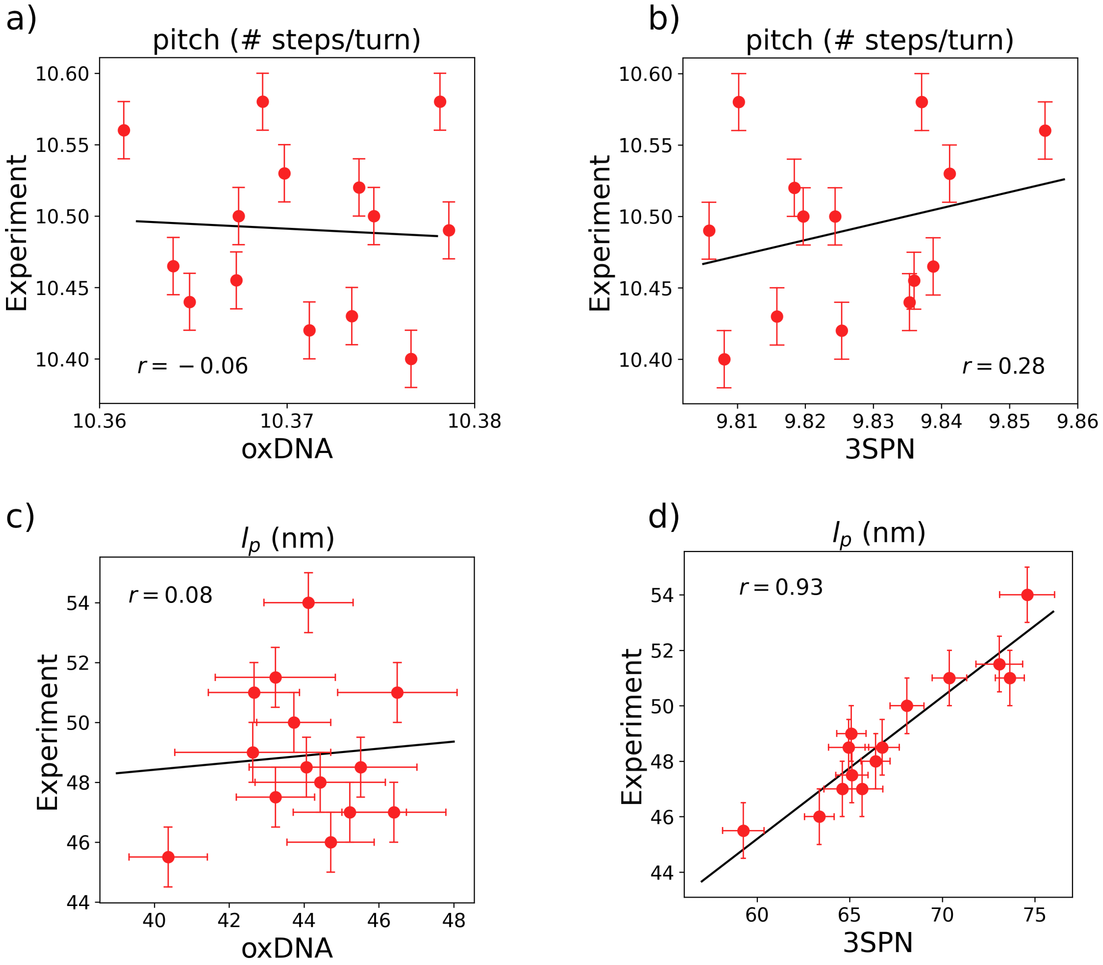

Figure S7: Comparison between experimental values and predictions by oxDNA2 and 3SPN2C for the sequence dependence of the helical pitch (a,b) and the persistence length l_p (c,d). The lines correspond to the linear fits of the scatter plots and are included as a guide to the eye. The value of the Pearson coefficient is reported in each plot.

S9 Convergence study of representative trajectories

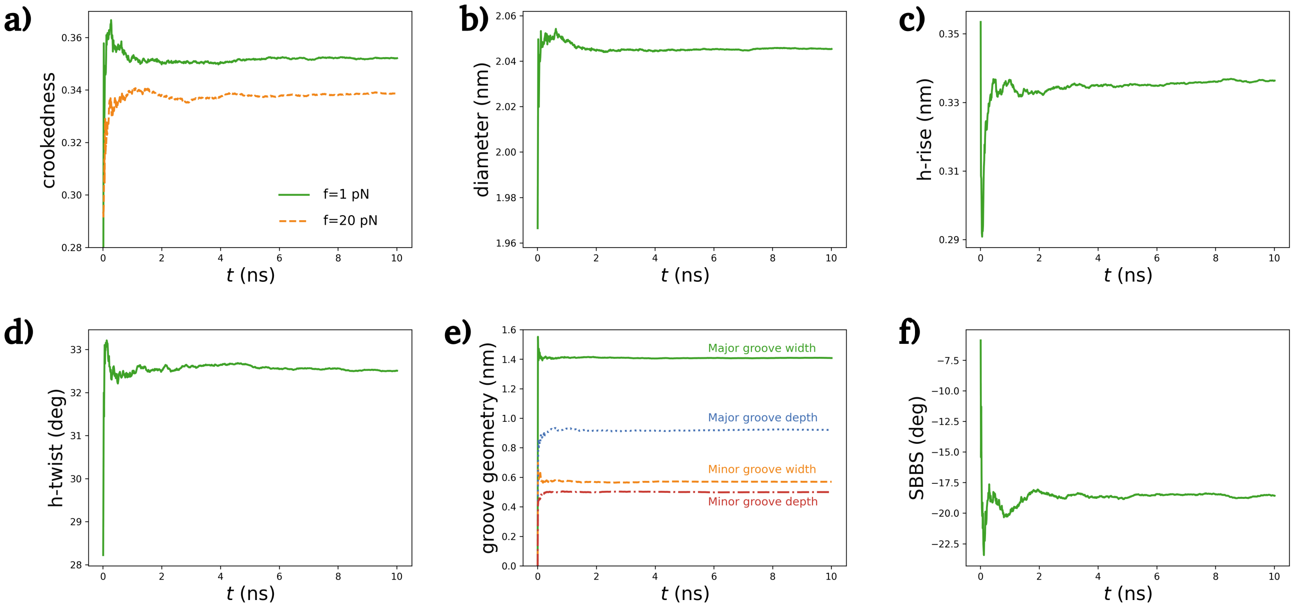

Figure S8: Convergence of the running average for various observables in a representative Benchmark simulation, corresponding to polyAA. In panels b)-f), we considered a simulation corresponding to $f = 1$ pN.

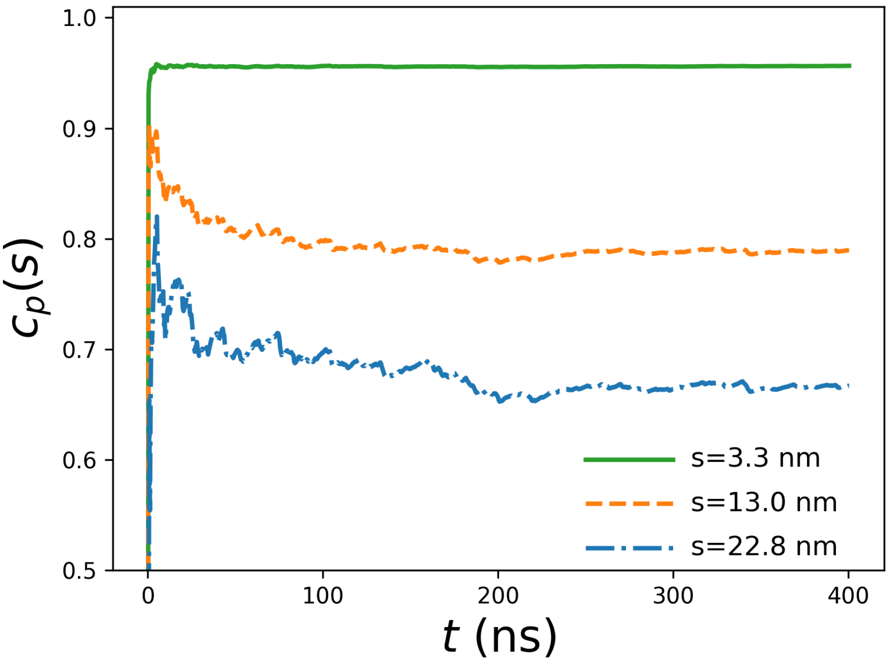

Figure S9: Convergence of the running average for the tangent vector correlation function $c_p(s)$ in a representative simulation for the computation of the sequence-averaged persistence length. We plot $c_p(s)$ for various values of the contour length s. The sequence considered in this representative case is P1.

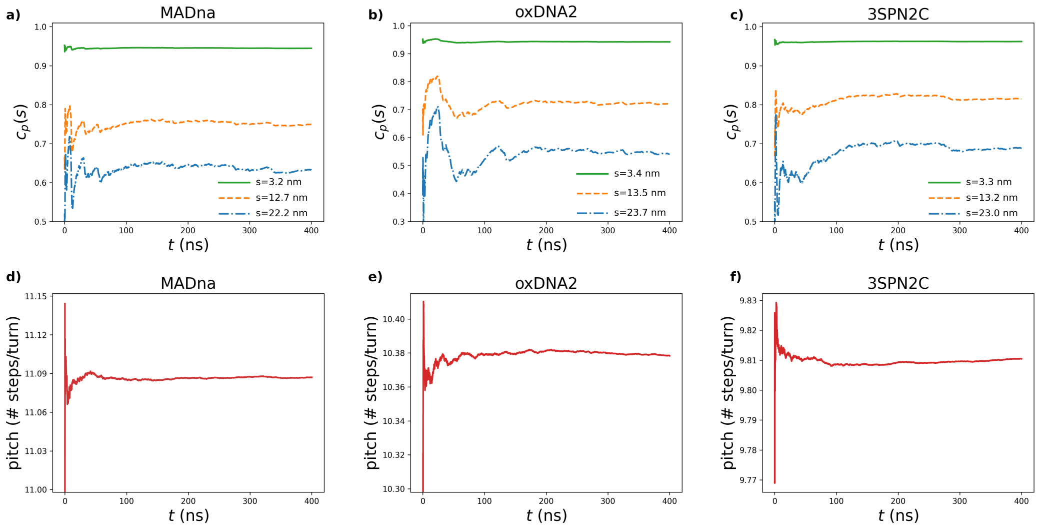

Figure S10: Convergence of the running average for the tangent vector correlation function $c_p(s)$ (top) and helical pitch (bottom) for a representative simulation for the computation of persistence length and helical pitch, for the three coarse-grained models. In the top panels, we plot $c_p(s)$ for various values of the contour length s. The sequence considered in this representative case is ACAT.

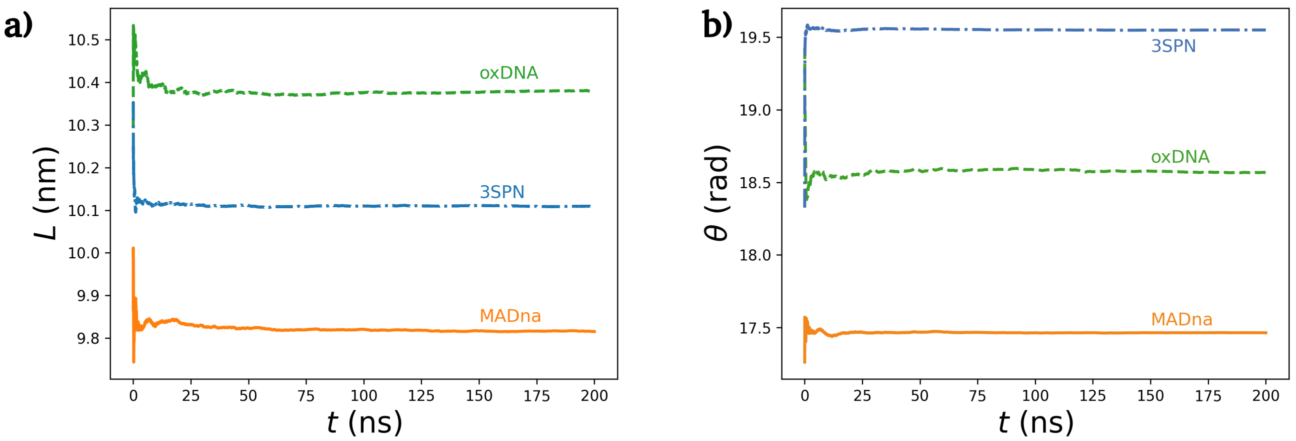

Figure S11: Convergence of the running average for the extension L (a) and cumulate h-twist θ (b) for the three coarse-grained models, for a representative trajectory corresponding to sequence ST1 under the action of a force $f = 2$ pN and a torque $\tau = 5$ pN·nm.

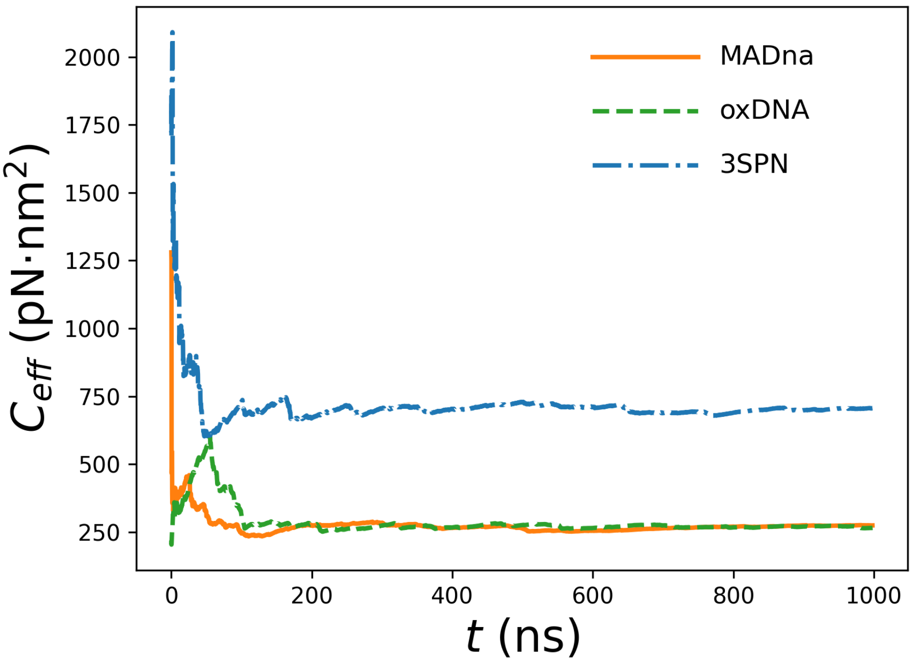

Figure S12: Convergence of the running average for effective twist modulus C_{eff} in a representative simulation for the study of the twist-bend coupling for the three coarse-grained model. The representative trajectory corresponds to a simulation performed for sequence TB1 at a force $f = 0.75$ pN.

Bibliography

[1] Hinckley, D. M., Freeman, G. S., Whitmer, J. K. and De Pablo, J. J. (2013) An experimentally-informed coarse-grained 3-site-per-nucleotide model of dna: Structure, thermodynamics, and dynamics of hybridization. *J. Chem. Phys.*, **139**, 144903.

[2] Marín-González, A., Vilhena, J. G., Moreno-Herrero, F. and Pérez, R. (2019) DNA Crookedness Regulates DNA Mechanical Properties at Short Length Scales. *Phys. Rev. Lett.*, **122**, 048102.

[3] Marín-González, A., Vilhena, J. G., Pérez, R. and Moreno-Herrero, F. (2017) Understanding the mechanical response of double-stranded DNA and RNA under constant stretching forces using all-atom molecular dynamics. *Proc. Nat. Academ. Sci.*, **114**, 7049–7054.

[4] D. A. Case, T. E. Cheatham III, T. Darden, H. Gohlke, R. Luo, K. M. Merz Jr, A. Onufriev, C. Simmerling, B. Wang amd R. J. Woods (2005) The Amber biomolecular simulation programs. *J. Comp. Chem.*, **26**, 1668–1688.

[5] A. Pérez, I. Marchán, D. Svozil, J. Sponer, T. E. Cheatham III, C. A. Laughton and M. Orozco (2007) Refinement of the AMBER force field for nucleic acids: improving the description of α/γ conformers. *Biophysical journal*, **92**, 3817–3829.

[6] M. Zgarbová, M. Otyepka, J. Sponer, A. Mládek, P. Banáš, T. E. Cheatham III and P. Jurecka (2011) Refinement of the Cornell et al. nucleic acids force field based on reference quantum chemical calculations of glycosidic torsion profiles. *J. Chem. Theory Comput.*, **7**, 2886–2902.

[7] W. D. Cornell, P. Cieplak, C. I. Bayly, I. R. Gould, K. M. Merz, D. M. Ferguson, D. C. Spellmeyer, T. Fox, J. W. Caldwell and P. A. Kollman (1995) A second generation force field for the simulation of proteins, nucleic acids, and organic molecules. *J. Am. Chem. Soc.*, **117**, 5179–5197.

[8] W. L. Jorgensen, J. Chandrasekhar and J. D. Madura (1983) Comparison of simple potential functions for simulating liquid water. *J. Chem. Phys.*, **79**, 926.

[9] I. S. Joung and T. E. Cheatham III (2009). *J. Chem. Theory Comput.*, **7**, 2886–2902.

[10] Plimpton, S. (1995) Fast Parallel Algorithms for Short-Range Molecular Dynamics. *J. Comp. Phys.*, **117**, 1-19.

[11] Mitchell, J. S., Glowacki, J., Grandchamp, A. E., Manning, R. S. and Maddocks, J. H. (2017) Sequence-Dependent Persistence Lengths of DNA. *J. Chem. Theory Comput.*, **13**, 1539–1555.

[12] Eberly, D. (2020) Least Squares Fitting of Data by Linear or Quadratic Structures. www.geometrictools.com/Documentation/LeastSquaresFitting.pdf

[13] Wing, R., Drew., H., Takano, T., Broka, C., Tanaka, S., Itakura, K. and Dickerson, R. E. (1980) Crystal structure analysis of a complete turn of B-DNA *Nature*, **287**, 755–758.

[14] Lu, X.-J. and Olson, W. K. (2003) 3DNA: a software package for the analysis, rebuilding and visualization of three-dimensional nucleic acid structures. *Nucleic Acids Res.*, **31**, 5108–5121.
